# Grit in second language acquisition: a systematic review from 2017 to 2022

**DOI:** 10.3389/fpsyg.2023.1238788

**Published:** 2023-09-01

**Authors:** Xian Zhao, Danping Wang

**Affiliations:** School of Cultures, Languages and Linguistics, The University of Auckland, Auckland, New Zealand

**Keywords:** grit, emotion, learning achievement, systematic review, positive psychology

## Abstract

Recent years have witnessed increasing attention to personality strength (grit) due to its merit in goal-seeking language learning processes. Two facets of grit, namely perseverance of effort (PE) and consistency of interest (CI), play a critical role in overcoming learning difficulties and strengthening willpower to pursue learning goals. The current review seeks to explore various issues related to grit, including its factor structure, the relationship between grit and frequently associated factors, as well as the utility of PE and CI in facilitating language learning. This exploration is based on the findings of 32 empirical articles published between 2017 and 2022 from three databases. The results indicate that although research which examines the role of grit has entered a fast growth phase since 2020, there is still a need for expansion and diversification in scopes, participants, research methods, and language contexts. Moreover, previous studies have not adequately addressed the critical issue of grit’s conceptualization and factor structure. Finally, this study suggests that future researchers should impartially assess the factor structure and nature of PE and CI, to provide more robust evidence to clarify the relationship between grit and diverse emotions and positive institutions, in order to advance understanding of grit in second language learning.

## 1. Introduction

The development of positive psychology in Second Language Acquisition (SLA) stimulate attention toward an array of non-cognitive factors in second language learning-related studies (MacIntyre and Mercer, 2014; Dewaele et al., 2019; Alamer, 2021; Wang et al., 2021). Theoretically established as one of the most significant pillars of positive psychology (Seligman, 2011), the role of personality strength (grit) in language learning has been gradually uncovered in recent years, though studies are few in number compared with other positive constructs. This increase in research has paved the way for further insights into the role of grit in facilitating language success (Credé and Tynan, 2021; MacIntyre and Khajavy, 2021; Sudina and Plonsky, 2021; Li and Yang, 2023).

Grit is firstly conceptualized as the “perseverance and passion” in the pursuit of long-term goals, and it had been initially featured as a higher-order construct that comprises two second-order latent facets, perseverance of effort (PE), and consistency of interest (CI; Duckworth et al., 2007, p. 1087). In other words, the success of grittier people depends on an essential blend of commitment and perseverance in pursuing a goal over a long period in spite of failures or obstacles. The process of second language/L2 learning is filled with intellectual and mental obstacles, necessitating the strength of grit to stimulate L2 learners’ continuous learning.

Grit scales have emerged and grown along with the turn toward positive psychology in the field of SLA. Duckworth et al. (2007) initially designed a 12-item grit scale (Grit–O). Duckworth and Quinn (2009) later refined this 12-item scale into a shorter eight-item version known as the short grit scale (Grit–S). Grit–S has been considered to have better psychometric properties than Grit–O, indicating its superiority when evaluating the effect of grit alongside other components (Duckworth and Quinn, 2009). Both scales have been widely adopted to measure language achievement across Chinese, Iranian, and United States contexts, though mainly in English language learning. However, these two grit scales have received some criticism due to the imbalanced gender ratio of participants and general domain contexts when gaging grit in language settings (Teimouri et al., 2020; Credé and Tynan, 2021). Recently, Teimouri et al. (2020) developed a nine-item domain-specific L2 grit scale which was subjected to principal component analysis that suggested the nine-item scale was superior to the 12-item scale as hypothesized by Duckworth et al. (2007). Alternatively, scale of L2–Grit of Alamer (2021) retained the 12-item structure which was evaluated using bifactor-confirmatory factor analysis. Methodologically, Alamer (2021) appears to be the first study to test the global factor structure of grit alongside the two specific factors of PE and CI factors.

Grit has gradually become popular in SLA due to its substantiated value for facilitating L2 learning (Lee and Drajati, 2019; Kiatkeeree and Ruangjaroon, 2022; Shehzad et al., 2022). A number of empirical investigations have supported the significance of grit in predicting L2 willingness to communicate (WTC; Lee and Hsieh, 2019; Lee and Lee, 2020; Lee and Taylor, 2022). In addition, grit appears to significantly and positively predict learning motivation, engagement, and English language achievement (Wei et al., 2019; Zhang et al., 2022; Zhao and Wang, 2023). The merits of grit have also been substantiated in the e-learning context (e.g., Liu et al., 2021).

Nevertheless, grit has received criticism in terms of its factor structure and predictive effect in SLA, which leads to questions concerning whether the promotion of its utility for L2 learning is reasonable (Credé and Tynan, 2021; Khajavy et al., 2021). In a recent meta-analysis, its original higher-order structure was not empirically validated, and grit was found to be only moderately associated with performance (Credé et al., 2017). The indirect effect of grit was confirmed in a longitudinal study by Alamer (2021), who claimed that grit components only indirectly predicted subsequent English vocabulary learning via the accumulation of PE and CI in the later learning phase. Further, some studies have suggested only PE significantly predicts L2 achievement across languages and contexts (e.g., Sudina et al. 2021; Khajavy and Aghaee, 2022), while others argued CI has merit in language success (e.g., Sudina and Plonsky, 2021; Alamer, 2022; Li and Yang, 2023). The incomplete understanding of the factor structure of grit, as well as the inconsistent evidence concerning the merit of PE and CI may lead to the misinterpretation of the function of grit. If so, language educators may fail to implement adequate measures to take advantage of L2 learners’ personality strengths to enhance their language achievement. In light of this, it is necessary to conduct a holistic exploration of the current development and issues surrounding grit in SLA to advance a nuanced understanding, identify future topics for researchers, and thus progress the field.

Therefore, this comprehensive literature review mainly addresses two research questions, the current state of grit research in SLA (RQ1) and current issues surrounding grit (RQ2). An overview of the current state of grit research including year of publication, author and their affiliation, research context and approach, research participant, research method, research design, research instrument, research tool, and target language. General uncertainties covering its factor structure, the frequent variables associated with grit, and the utility of PE and CI in SLA are then examined. To achieve this, we collected 32 articles published from 2017 to 2022, a period of rapid growth for grit research in SLA, according to our inclusion and exclusion protocol to elucidate existing concerns and suggest potential directions for future research.

## 2. Methodology

### 2.1. Systematic review

Systematic review develops thorough and visible procedures, specifically determining the search terms, selecting the search databases, implementing inclusion and exclusion criteria, screening studies, and reporting results (Zawacki-Richter et al., 2020). It offers an opportunity for scholars better to familiarize themselves with a topic from a holistic perspective, pinpoint existing gaps, and formulate hypotheses based on prior studies. Thus, in order to gain a nuanced understanding of grit in terms of its current state, factor structure, association with frequently related constructs, and the separate role of PE and CI in language learning from the perspective of positive psychology, this study implemented a systematic review approach to search and select data from research published between 2017 and 2022. We collected articles by searching the key terms “grit” and “language learning” within three sizable databases (Scopus, Web of Science, and Eric), which contain the greatest quantity of studies relating to education (Wang and Tsung, 2022).

### 2.2. The inclusion and exclusion criteria of the current study

We initially identified 225 articles from three databases. Duplicated resources (*N* = 42) and literature not published between 2017 and 2022 (*N* = 12) were eliminated from the database. The remaining publications (*N* = 171) were further examined based on the inclusion and exclusion criteria of our systematic review to determine the scope and ensure the quality of the included database. For inclusion, studies should be: peer-reviewed journal articles; written in English; and examine both grit and language learning achievement in the L2 learning context. Based on these criteria, some articles (*N* = 103) were eliminated due to their inability to fulfill the criteria as indicated by the title, abstract, and methodology section.

- Not peer-reviewed journal articles (book chapters, conference papers, doctoral dissertations, patents, meetings, datasets, and other non-identified formats; *N* = 85).
- Not English-language publications (written in other languages; *N* = 5).
- Not examining grit and language learning achievement in the language learning context (off-topic; *N* = 13).

After discarding the non-conforming articles in the first round, 68 research articles remained in the data set. Subsequently, an extra round of inclusion and exclusion criteria was implemented by thoroughly and systematically reading the full text to avoid missing important information. In addition to the first set of criteria, we determined that, for the final analysis, articles should be: categorized as quantitative or mixed-method studies; have students as participants; recruit over 100 participants to satisfy the criteria of the minimum sample size for factor analysis and to improve the generalizability of findings; and include pedagogical implications based on the empirical results of the studies. Therefore, some articles (*N* = 36) were disqualified because they did not meet these requirements.

- Not quantitative studies (systematic review, meta-analysis, theoretical analysis, and qualitative studies; *N* = 28).
- Not having students as participants (the focus was on teachers; *N* = 2).
- Not having an adequate number of participants (*N* < 100; *N* = 4).
- Less focus on educational outcomes (only focus on developing instruments; *N* = 2).

**Figure 1:**
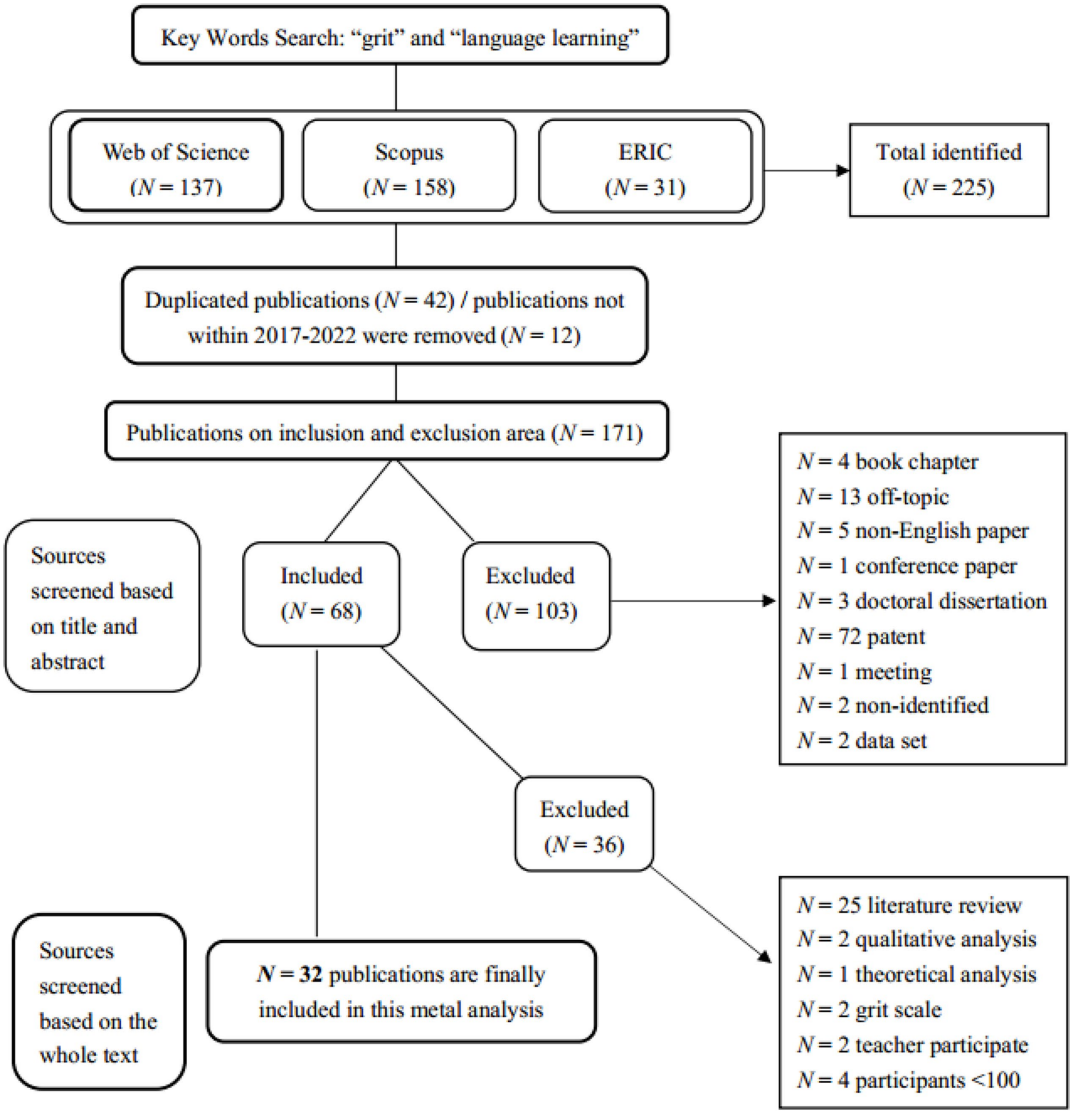
Flowchart of the inclusion and exclusion process.

The process of data selection was carried out in September 2022. After employing the two rounds of assessment for inclusion, based on the criteria mentioned above, we identified 32 publications that qualified for further systematic review. Figure 1 illustrates the detailed process that was designed based on a paradigm from a similar topic in the field (Christopoulou et al., 2018).

## 3. Results

The 32 studies of this systematic review addressed two research questions proposed by this study. The results fall into four sections concerning each research question. To understand the current state of grit research in L2 learning (RQ1), information covering the year of publications, author(s) and their affiliations, research contexts and teaching approaches, research participants, research methods, research designs, research instruments, research tools, and target languages were presented and classified. To gain a nuanced understanding of the current issues of grit (RQ2), we subsequently reviewed findings regarding its factor structure, the frequent variables associated with grit, and the utility of PE and CI in L2 learning.

### 3.1. The current research state of grit in L2 learning

This study has identified a number of features from the 32 documents gathered (see Table 1). With regard to the publication timeline, four studies were published in 2019, six in 2020, five in 2021, and 17 were published in 2022. No articles published in 2017 and 2018 were eligible for inclusion. We also found that male researchers (*N* = 26) outnumbered their female counterparts (*N* = 6). For university affiliation, the top authors are Lee Ju Seong (six articles) from the Education University of Hong Kong, Abdullah Alamer (two articles) from King Faisal University, Gholam Hassan Khajavy (two articles) from the University of Bojnord, Ekaterina Sudina (two articles) from Northern Arizona University, and Faramarz Ebn-Abbasi (two articles) from Shahid Beheshti University. Researchers from Asian and Middle Eastern countries/regions have conducted the majority of the grit research.

**Table 1:** General characters of reviewed studies (*N* = 32).

First author	Number	Sample source	Number
China	11	Chinese	12
South Korean	6	Iran	7
Iran	6	Saudi Arabia	4
Saudi Arabia	3	Korea	3
The United States	2	International sample	2
Pakistan	1	Thailand	2
Thailand	1	Indonesia	1
Australia	1	The United States	1
Poland	1		
Teaching context	Number	Language	Number
In class	29	English	31
Out of class	3	Other	1
Research method	Number	Research question	Number
Quantitative	28	Correlation between grit and other factors	28
Mixed-method	4	Factor structure/conceptualization	8
Qualitative	0	Source of grit	1
Education context	Number	Instruments (adopted or adapted)	Number
University	24	Grit–S (Duckworth and Quinn, 2009)	12
Mixed	3	L2 grit (Teimouri et al., 2020)	11
High	3	Grit–O (Duckworth et al., 2007)	4
Junior	1	Other	3
Primary	1	L2 grit scale (Alamer, 2021)	2
Study design	Number	Analysis method	Number
Cross-sectional	30	Regression analysis	19
Longitudinal	2	SEM/ESEM	12
		Other	1

In addition, 90.6% of publications were related to traditional L2 learning situated in classroom, with the remaining small portion of research examining L2 learning outside of class. Participants’ educational backgrounds ranged from primary school to university level, though the majority of studies focused on university learners (primary school: 3.1%; junior high school: 3.1%; senior high school: 9.4%; mixed participants: 9.4%; and university: 75%). The participants came from Chinese contexts (including Hong Kong and Taiwan, *N* = 12), Iran (*N* = 7), Saudi Arabia (*N* = 4), Korea (*N* = 3), Thailand (*N* = 2), Indonesia (*N* = 1), the United States (*N* = 1), and international samples (*N* = 2), with the sample size ranging from 154 to 1,265. One of the exclusion criteria is that the participant sample size must be above 100 in order to conform to ecological validity. The majority of participants in the included peer-reviewed studies were female.

With regard to methodologies, 30 studies (93.94%) were cross-sectional, and two were longitudinal (Alamer, 2021, 2022). The majority of reviewed articles were quantitative research, with only four being mixed-method studies. Twenty studies utilized regression analysis performed by SPSS software and 12 utilized structural equation models computed using Amos or Mplus.

In terms of the target language, 31 articles examined English as L2 learning except for one article which focused on Spanish and French language learning (Sudina and Plonsky, 2021). This indicates that grit-related research requires further attention to and exploration of languages other than English to broaden the research scope and obtain a more fulsome picture of grit in the field.

### 3.2. The factor structure of grit

Among reviewed publications, we found that the eight-item general Grit–S scale (Duckworth et al., 2007) or the eight-item Grit–O scale (Duckworth and Quinn, 2009), which were adopted by the largest number of studies (*N* = 16). Eleven publications adopted or adapted the nine-item domain-specific L2 grit scale (Teimouri et al., 2020), which was well-featured in studies from the previous 2 years, indicating a tendency to employ or develop a novel scale to better reflect learners’ continuous effort and consistent interest in the specific L2 context. Additionally, it is worth mentioning that 12-item L2grit scale of Alamer (2021) is the first study to test the global factor of grit alongside the two specific factors of PE and CI factors using sophisticated bifactor-confirmatory factor analysis.

Nevertheless, only eight articles officially proposed a factor structure for grit as one of their primary research questions. The results of Alamer (2021) and Sudina et al. (2021) were identified statistically as being consistent with the original conceptualization of grit and instrument findings of Duckworth et al. (2007) and Sudina et al. (2021) showed that L2 grit was a second-order two-dimensional structure in English language learning contexts among the international sample. The two components were moderately associated in the L2 context but were highly correlated in the foreign language context. In contrast, in the Saudi Arabian context, Alamer (2021) adopted and validated a 12-item L2 grit scale based on the Grit–S (Duckworth et al., 2007) and Academic Grit Scale (Clark and Malecki, 2019). The results indicated that grit was a single construct with two specific scales, as initially conceptualized by Duckworth et al. (2007).

Of interest was the finding that no consistent evidence was detected by Khajavy et al. (2021) in the Iranian context in their validation of the Grit–S instrument with university English learners. The result contrasted with previous considerations of grit, as having a higher-order unitary structure by demonstrating the hypothesized first-order two-factor structure of grit fit the data better than the single-factor structure.

The L2 grit scale developed by Teimouri et al. (2020) underscored the domain specificity of grit and attracted increasing attention in the area of L2 learning. Situated in the Iranian context and confirmed the existence of two significant components, Teimouri et al. (2020) found that the L2 grit scale yielded a stronger predictive power than a domain-general scale. It was established that grit and conscientiousness are conceptually different, in contrast to Duckworth’s opinion that grit “overlap[s] with achievement aspects of conscientiousness” (p.1089). By further validating the L2 grit scale in Taiwanese college English major students, Cheng (2021) argued grit is a two-factor model comprising two interrelated yet unique subcomponents (PE and CI) in the Chinese context.

In a sample of Spanish and French foreign language learners in the United States, Sudina and Plonsky (2021) suggested grit is a higher-order construct encompassing PE and CI (distinct but related factors) by validating the L2 grit scale. The relationship between PE and CI was stronger than the result reported by Teimouri et al. (2020). Because the promotion of personality strength (grit) in positive psychology is still in its infancy, sufficient empirical studies concerning its definition and instrument are needed to ascertain if the value of grit is plausible in L2 learning research.

### 3.3. Grit, associated variables, and L2 learning achievements

Grit has been frequently linked to academic attainment, L2 WTC, and emotion in the educational context (*N* = 17). Encompassing grit and WTC, the bulk of empirical studies confirm a higher level of grit is followed by a higher degree of L2 WTC in the classroom among Korean (Lee and Lee, 2020; *N* = 176) and Indonesian students at the university level (Lee and Drajati, 2019; *N* = 176). Beyond traditional classes, grit was positively linked with L2 WTC across the traditional, digital, and distance online classrooms reflected by Taiwanese non-English undergraduates (Lee and Hsieh, 2019; *N* = 261). In a recent study, classroom enjoyment, grit, and extracurricular English are altogether significant predictors of classroom L2 WTC for primary school L1 Cantonese speakers in Hong Kong (Lee and Taylor, 2022; *N* = 160).

In contrast to the abovementioned findings, however, some studies reported an indirect or weak effect of grit in L2 learning. For example, grit mediated the association between the ideal L2 self and L2 WTC based on 842 undergraduate English major students (Lan et al., 2021). Cheng (2021) reported both grit components significantly but moderately or weakly predicted the WTC of Taiwanese college students (*N* = 294). A critical longitudinal study conducted by Alamer (2021) found that grit was not a significant predictor of English vocabulary learning, and initial grit only indirectly influenced vocabulary knowledge through the accumulation of later grit spanning approximately one academic year (*N* = 154).

Nevertheless, the value of grit in predicting L2 achievement appears to have reached a consensus across Chinese and Thai contexts. For instance, grit significantly and positively predicted learning motivation, academic engagement, and English performance among Chinese students at university (Wu et al., 2022; *N* = 624; Zhang et al., 2022; *N* = 493) and middle school levels (Wei et al., 2019; *N* = 832), which has also been reported with high school participants in Thailand (Kiatkeeree and Ruangjaroon, 2022; *N* = 563). Additionally, low grit appears to reduce English as foreign language/EFL learners’ academic efficacy in China under the mobile e-learning context (Liu et al., 2021; *N* = 554).

Some inconsistent results were found in the Middle Eastern context. For example, in the only longitudinal study based on the Saudi context, Alamer (2021) stated there were no statistically significant effects of grit, CI, or PE on later English vocabulary learning, yet grit subsequently played a role as a mediator in the model. Utilizing the structural equation model approach, Khajavy et al. (2021) reported no relationship exist between grit (both PE and CI) and university students’ L2 achievement in Iran (*N* = 1,178), and Khajavy and Aghaee (2022) argued only PE significantly predicted L2 achievement if grit was the only predictor in the model. Concerning the power of CI and PE, further empirical results will be reviewed in the section 3.4.

In addition, 15 studies combined grit with emotion in English language learning. Of all constructs, the two emotions most typically associated with grit were foreign language enjoyment (*N* = 10) and anxiety (*N* = 9), which has been considered to be the “left and right feet” that carry L2 learners on their way to success (Dewaele and MacIntyre, 2014). There was a consensus in the reviewed studies that positive correlations were found between positive emotions and grit, and vice versa. In studies in the Chinese context, Liu and Wang (2021), Wei et al. (2019), and Yang et al. (2022) found grit significantly correlated with enjoyment among middle school, high school, and university learners, which was also reported for Iranian English learners (Hejazi and Sadoughi, 2022; *N* = 339; Pawlak et al., 2022; *N* = 238). Khajavy and Aghaee (2022), Liu and Wang (2021), and Liu et al. (2021) found that grit negatively correlated with English learning anxiety. Liu and Wang (2021) indicated the mediating effect of anxiety was significantly stronger than FLE on the relationship between grit and EFL achievement. Boredom, shyness, and burnout appeared once each in the reviewed articles. Surprisingly, L2 boredom was positively predicted by CI, which necessitates cross-validation in future empirical studies (Pawlak et al., 2022). Selecting a less featured negative emotion, Lan et al. (2021) found shyness was negatively associated with grit in a mediate moderate model across Chinese English major students at the university level. The relationship between grit and other less-featured emotional constructs need to be further substantiated.

### 3.4. The merit of PE and CI in L2 learning

Twelve publications explored the two dimensions of grit by associating it with other constructs from a positive psychological perspective in English language learning. However, there exists some divergence in terms of the power of PE and CI when they were connected with other constructs across different contexts, participants, educational backgrounds, and the involvement of other components.

Most studies indicated that PE and CI significantly predicted EFL learning performance and success across China, Iran, and Saudi Arabia. Alamer (2021) observed that despite the initial predictive effect of PE, CI, and grit, their impact on subsequent English vocabulary learning was not statistically significant. Nevertheless, the indirect effect of initial grit components was statistically significant due to the durability of both PE and CI in later English learning. Analogously, Teimouri et al. (2020) found both PE and CI were positively correlated with joy but negatively correlated with anxiety in L2 English learning, yet PE had a much stronger association with motivational and emotional factors than CI based on 191 L1 Persian students at a private Iranian university. The result was in line with Cheng (2021), who reported that both PE and CI significantly predicted L2 WTC in a sample of 294 English major university students in Taiwan, but PE generated a more substantial predictive effect than CI. Shehzad et al. (2022) specified the relationship between grit (PE and CI), self-efficacy beliefs toward pronunciation, and pronunciation performance based on 350 Saudi Arabian EFL university students. They found that both CI and PE significantly and positively predicted L2 learners’ pronunciation performance and self-efficacy beliefs. Further, by quantifying the correlation between emotion, grit, and motivation behavior in EFL learning among 238 Iranian university students, Pawlak et al. (2022) suggested that both PE and CI mediated the relationship between enjoyment and motivation behavior, and that enjoyment significantly and positively predicted both PE and CI.

Other studies indicated inconsistent findings in terms of the predictive role PE and CI in L2 learning. On the one hand, the majority of empirical studies consider PE to be a superior facet, indicating a lesser importance of CI in facilitating L2 learning. For instance, Sudina et al. (2021) suggested PE, along with classroom anxiety, rather than CI, was a significant predictor of self-reported English proficiency based on international participants (*N* = 454). In the Saudi Arabian context, Elahi Shirvan and Alamer (2022) reported that only PE was positively linked with L2 achievement, and PE indirectly mediated the effect between competence and L2 achievement among undergraduate students (*N* = 213). Similarly, Lee (2022) found only PE and enjoyment were predictors of all Korean cohorts’ L2 WTC (middle school: *N* = 137; high school: *N* = 323; and university students: *N* = 187). By comparing the strength of L2 grit (PE and CI) and learning motivation on L2 WTC among Iranian EFL learners across high school and private institutes, Ebn-Abbasi et al. (2022) demonstrated only PE and classroom engagement, except for CI, exerted a significant effect on L2 WTC in both educational settings (*N* = 308). A recent article by Khajavy and Aghaee (2022) reported that when other predictors were included in the structural equation model, only PE indirectly predicted L2 enjoyment and personal best goals reflected by Iranian English learners (*N* = 226), indicating a mix of other factors might interfere with the function of grit as originally conceived. On the other hand, other empirical studies revealed that CI has merit in catalyzing L2 learning success. Sudina and Plonsky (2021) reported that CI and FL buoyancy were the strongest positive predictors of language achievement in their cross-sectional research of 360 FL Spanish (*N* = 258) and French (*N* = 102) students in the United States, which is different from previous results (Sudina et al., 2021). By employing the Exploratory Structural Equation Modeling analysis in a longitudinal study, Alamer (2022) further claimed that single language interest indirectly predicted English achievement spanning approximately one academic year. Several studies indicated PE and CI do not appear to be predictors of learning achievement. For example, Khajavy et al. (2021) failed to determine the correlations, if any, between PE, CI, and EFL learning achievement among Iran university learners in the structural equation model. Such inconsistency warrants further explorations across languages, contexts, participants, models, along with the interaction with diverse variables.

## 4. Discussion and implications

The current study, based on 32 research articles published between 2017 and 2022 from three databases, investigated the current state of grit-related research (RQ1) and the most commonly investigated issues (RQ2) in L2 learning. These empirical attempts are critical as they are highly significant to the rationale for further exploration of grit in SLA. In view of this, four suggestions for future research in this area can be drawn from the collection of the reviewed publications.

### 4.1. Status quo of current research

The progress of positive psychology in SLA witnessed a rapid growth of grit research since 2020. A substantial number of studies have tapped into the conceptualization, measurement, and merit of grit across different perspectives such as learning contexts, research scopes, methods, and participants.

The majority of grit research focuses on L2 learning in traditional face-to-face learning contexts (Wei et al., 2019; Khajavy et al., 2021; Kiatkeeree and Ruangjaroon, 2022), which has led to an incomplete understanding of the nature of grit as different learning contexts may shape the utility and predictive effect of grit. Therefore, to provide further insights in the post-pandemic era, future studies could consider focusing on online remote L2 learning, blended learning, and mobile learning not only because of their own distinctive features but also because these delivery modes represent new trends in education. In addition, it is essential to tailor and adapt pedagogical approaches across different teaching contexts to better exploit the merit of grit and enhance L2 learners’ learning achievement (Li and Dewaele, 2021). Comparative and experimental studies examining online and offline learning, and in-class and out-of-class learning are also necessary to gain a better understanding of grit.

In addition, researchers from Asian and Middle Eastern countries and regions appear to be more interested in grit than researchers from other areas, and current literature primarily focuses on English language learning. Only one study focused on languages other than English, namely, learning Spanish and French as a FL (Sudina and Plonsky, 2021). The lack of grit research in other languages might hinder its development in this field due to an inadequate understanding. To address this, future research should expand the scope and examine the role of grit across diverse languages and move beyond Asian and Middle Eastern contexts to obtain a better understanding concerning the function of grit in SLA.

Furthermore, in terms of the research methods, the number of quantitative, qualitative, and mixed-method studies in this area is unbalanced, being dominated by self-report cross-sectional studies (29 in total), which indicates an absence of in-depth qualitative and mixed-method studies (Lan et al., 2021; Sudina et al., 2021; Shehzad et al., 2022; Wu et al., 2022). Further investigations could expand on current knowledge in the following two ways. The qualitative or mixed method approach, instead of pure data-driven quantitative research, is needed as it is conducive to allowing further articulation of antecedents, consequences, and the complex networks of personality strength in L2 learning (Lee and Taylor, 2022). In addition, longitudinal studies with both longer and shorter time frames are required to ascertain the durability and malleability of grit with regard to language achievement (Alamer, 2021).

Finally, current research mainly focuses on foreign language learners at the tertiary level, neglecting the consideration of younger learners and constraining the generalizability of prior results and pedagogical implications. The consideration of the learning perseverance and passion of adolescent learners is beneficial to the development of their language skills in the future as they are at a critical stage in L2 learning. In addition, social, economic, and demographic variables may impact learners’ psychological states and learning behaviors in L2 learning. Thus, understanding whether and how grit is influenced by age, gender, and educational background across different populations in L2 learning may uncover further implications.

### 4.2. The factor structure issue

An explicit conceptual structure and instrument with acceptable reliability and validity are critical prerequisites for conducting a study (Elahi Shirvan et al., 2021). However, the factor structure and measurement of grit have long been a controversial issue that has yet to be fully addressed.

In terms of the conceptual structure of grit, future studies would be more constructive and rigorous if researchers could refine the conceptualization of grit. There are three parts embedded in its definition, which are investing continuous effort and maintaining interest no matter what obstacles and difficulties may be encountered in pursuing long-term goals. However, to our knowledge, few current studies have provided a well-defined meaning for ‘long-term goal’ nor addressed difficulties in the definition of grit (Credé and Tynan, 2021; MacIntyre and Khajavy, 2021). A recent important attempt by Alamer (2022) deepened the understanding of the role and conceptualization of CI in L2 English learning by offering the term “autonomous single language interest,” which necessitates further consideration of the quantity of interests and the degree to which they are internalized. Nevertheless, this is a significant advance in better conceptualizing grit in the discipline. Redefining the grit framework and embracing the domain-specific approach in the L2 context is vital to clarify the conceptualization and function of grit in L2 learning (Teimouri et al., 2020; MacIntyre and Khajavy, 2021; Li and Yang, 2023). In addition, few current studies have differentiated grit from other overlapping elements (i.e., conscientiousness), which may obstruct a deeper understanding of grit (Khajavy and Aghaee, 2022).

Regarding the current two commonly adopted instruments (Grit–S and the L2 grit scale), we firstly reiterate that the validity and reliability of Grit–S (Duckworth and Quinn, 2009) and the L2 grit scale (Teimouri et al., 2020) in the SLA context are not without flaws (Credé and Tynan, 2021; Wang et al., 2021), and they require further improvement. It is risky to uncritically adopt any grit scale when exploring the role of grit in any educational setting (Zhao and Wang, 2023). However, researchers often adopt these scales in empirical studies without first statistically examining its factor structure, which might result in continued questions about its applicability and validity.

Therefore, validate and extend both popular scales across different languages and contexts are further warrented (Zhao and Wang, 2023). Additionally, as the cross-sectional studies fail to capture possible dynamic features of grit, future studies could explore the measurement invariance of the grit scale across populations and time windows. Validating the instrument by utilizing the “analysis-curve of factors model (a longitudinal confirmatory factor analysis-curve of factors model/LCFA-CFM approach)” in the longitudinal study is also needed (Elahi Shirvan et al., 2021, p.7). Furthermore, it would be more comprehensive and scientific if researchers could incorporate qualitative data when conceptualizing grit (Alamer, 2022). Validating the scale to the under-explored outdoor setting, e-learning or self-learning context is of great importance. New instruments need to be developed and validated according to the needs of different contexts.

### 4.3. The association between grit and other factors

Grit has been considered an essential personality strength that catalyzes language growth, which could not be easily attained with only talent (Duckworth et al., 2007). Nevertheless, it appears that the merit of grit in enhancing L2 English learning has yet to reach a consensus across contexts, not to mention in languages other than English. To obtain a fuller picture of the strength of grit in L2 learning, future studies should explore grit across different cultural contexts to provide tailored pedagogical insights for educators and learners.

Moreover, the existing research mainly concerns the relationship between grit and two common emotions, enjoyment and anxiety (Liu and Wang, 2021; Yang, 2021; Hejazi and Sadoughi, 2022), which has led to an incomplete understanding of the role of grit in language learning. In recent years, with the progress of positive psychology, the negative effect of boredom has been gradually acknowledged in hindering language learning success. However, only a handful of studies have examined the relationship between grit and other emotions in predicting L2 achievement (Lan et al., 2021; Pawlak et al., 2022; Zhao and Wang, 2023). Building on the “three pillars” of positive psychology (Seligman, 2011), the combination of personality strength and emotion are intertwined to contribute to an individual’s growth (Lee and Lee, 2020; Sudina et al., 2021; Yang, 2021). Therefore, more robust empirical evidence is required to advance the relationship between grit and emotions in the language learning mechanism, for example, boredom, love, happiness, shyness, anger, and disappointment. Further, a necessary future direction for research would be to associate grit with both emotion and the third pillar of positive psychology (positive institution) to advance the complex relationship among those three pillars.

### 4.4. The separate role of PE and CI in L2 learning

It is worth commending that increasing numbers of researchers have gained awareness about the unequal utility of grit components in facilitating L2 learning (Teimouri et al., 2020; Sudina et al., 2021; Khajavy and Aghaee, 2022). However, researchers and educators should recognize that CI is a contributing factor in L2 learning, as reported by previous studies. For example, Alamer (2022) substantiated the dynamic and indirect role of CI in enhancing language success in the long run in the Saudi Arabia context. Li and Yang (2023) and Zhao and Wang (2023) confirmed the role of grit components in German and English language achievement among Chinese learners. There is a potential risk, for example, that the swift change in focus to PE and the eschewing of CI in measuring academic success (Credé et al., 2017) may lead to an incomplete and unbalanced understanding of the utility of grit. As a non-cognitive factor, grit study has received attention in L2 learning in recent years, yet understanding the separate role of PE and CI in educational outcomes is still in its infancy. Hence, it is important to scrutinize the role of PE and CI in an unbiased manner and provide sufficient evidence of prediction of learning achievement prior to any decision to discard either component of grit.

Notably, only a few studies identify the factor structure of grit (Khajavy and Aghaee, 2022; Li and Yang, 2023; Zhao and Wang, 2023), with the bulk of studies either neglecting to examine the validity of grit or mistakenly separating grit after confirming the original higher-order two-factor structure of grit. Therefore, in our view, future studies should understand that the separation of the combined grit subcomponents depends on statistical examination based on the study’s participants and contexts, rather than instinctive decision-making. That is, researchers should adopt confirmatory factor analysis to carefully investigate the factor structure of grit. If the statistical results indicate it is a one-factor structure, we suggest that grit could be considered an integral component. Otherwise, it is highly recommended to further examine the specific merits of the two sub-components in the mechanism.

## 5. Conclusion and limitations

As a non-cognitive factor, personality strength (grit) has long been eschewed in the field of SLA in favor of the promotion of the significance of cognitive factors (Li and Dewaele, 2021; Alamer, 2022; Khajavy and Aghaee, 2022). However, as a pillar of positive psychology, grit plays an important role in promoting language success (Seligman, 2011; Teimouri et al., 2020). This study utilized a systematic review approach to include and exclude papers published between 2017 and 2022 from threedatabases.

This systematic review revealed four major findings. Above all, current empirical studies are mainly situated in China, Iran, Korea, and Saudi Arabia, indicating the necessity of the expansion of future research in terms of the scopes, research sites, and participants, in addition to comparative studies which use different teaching modes in SLA. Furthermore, previous studies are dominated by the data-driven approach. Future research could consider conducting qualitative and mixed-method studies to explore the antecedents and results of grit to provide effective teaching interventions. Additionally, the concept of grit needs to be further refined to make it clear and measurable, and validate the grit measurements across contexts. Furthermore, the investigation of grit and diverse emotions from a holistic perspective in the mechanism would be greatly needed. Lastly, despite a bulk of studies suggesting that PE has a better utility than CI, recognition of the importance of CI is gradually increasing. The abandonment of any sub-components of grit cannot yet be decided upon.

This study is one of the few attempts to offer a timely and systematic L2 grit review for readers and researchers to facilitate their understanding of this popular topic, identify potential issues, and suggest potential solutions to move the field forward. Specifically, providing researchers with more solid foundations for conceptualizing and assessing grit in L2 learning, and identifying further concerns and future trends when conducting grit-related studies. However, this study is not free from limitations that may restrict the generalizability of its findings. Firstly, although this study searched multiple databases with the highest number of studies concerning language learning and teaching in educational settings, it would be more comprehensive if it included studies from more diverse databases. Secondly, the selection criteria employed excluded several doctoral dissertations, which may have meant some important insights were lost. Finally, the sources of grit and the influence of demographics on grit still require further attention.

## Author contributions

All authors listed have made a substantial, direct, and intellectual contribution to the work and approved it for publication.

## Funding

This work was supported by China Scholarship Council (CSC) (Grant No. 202208250024).

## Conflict of interest

The authors declare that the research was conducted in the absence of any commercial or financial relationships that could be construed as a potential conflict of interest.

## Publisher’s note

All claims expressed in this article are solely those of the authors and do not necessarily represent those of their affiliated organizations, or those of the publisher, the editors and the reviewers. Any product that may be evaluated in this article, or claim that may be made by its manufacturer, is not guaranteed or endorsed by the publisher.
